# Comment on “Independent associations of high‐density lipoprotein cholesterol and triglyceride levels with Alzheimer's disease and related dementias”

**DOI:** 10.1002/alz.70233

**Published:** 2025-05-23

**Authors:** Fangbo Lin

**Affiliations:** ^1^ Rehabilitation Medicine Department The Affiliated Changsha Hospital of Xiangya School of Medicine, Central South University (The First Hospital of Changsha) Changsha China; ^2^ Neurology Department Fujian Medical University Union Hospital Fuzhou China

1

Dear Editors,

We read with great interest the article titled “Independent associations of high‐density lipoprotein cholesterol and triglyceride levels with Alzheimer's disease and related dementias” published in *Alzheimers & Dementia*.^1^ The intriguing finding that low levels of both high‐density lipoprotein cholesterol (HDL‐C) and triglycerides (TGs) were associated with an increased risk of Alzheimer's disease and related dementias (ADRD) merits further discussion, especially from a clinical standpoint. I would like to raise several points of concern and clarification.

First, the baseline characteristics reveal notable heterogeneity in age, ethnicity, and comorbid conditions (e.g., cardiovascular disease). Although the authors adjusted for age, sex, and body mass index via residualized HDL‐C and TG values, additional confounding factors—especially frailty and preclinical conditions—could contribute to altered lipid levels and thus modify dementia risk. Clinically, older adults with low levels of HDL‐C or TGs might also be more likely to have undiagnosed malnutrition or cachexia, conditions that may predispose them to cognitive decline through alternative pathways.^2^

Second, the temporal stability of HDL‐C and TG levels is not clearly addressed. In clinical practice, lipid profiles may fluctuate with changes in diet, medication use (statins, fibrates, niacin), and comorbid diseases. A single baseline measure might not adequately capture the longer‐term lipid exposure relevant to ADRD pathogenesis. More frequent longitudinal assessments of lipid levels could yield deeper insights into whether persistent, rather than transient, lipid alterations influence dementia risk.

Third, the potential impact of lipid‐altering therapies also deserves attention. Individuals with particularly high or low HDL‐C or TG levels may have undergone more aggressive treatment, which could alter their baseline risk through pathways unrelated to lipids per se (e.g., improved vascular health or better overall medical follow‐up). Clarifying the role of these medications in moderating ADRD risk would be valuable, especially given the growing emphasis on the intersection of vascular and neurodegenerative pathologies.

Fourth, given the observed protective association of higher TG levels, it would be illuminating to explore interactions with other cardiometabolic markers, such as insulin resistance, glycemic control, or subclinical inflammatory markers. A complete cardiometabolic profile might help disentangle the biologic processes that link lipid abnormalities to cognitive decline. High TG levels might, in some cases, reflect underlying insulin resistance—an important contributor to vascular and metabolic risk—although it seems from these findings that elevated TG level alone, in the context of typical body mass and other clinical factors, could be protective.^3^ This nuanced interplay merits further investigation.

Finally, the authors’ use of residualized HDL‐C and TG values (conditional on age, sex, and body mass index) is statistically valid, but may limit the direct applicability of these findings to everyday clinical settings, where clinicians rely on standard lipid measurements. Additional discussion on how these “residualized” values translate to routine lipid panel interpretations would aid in bridging the gap between research and practice.

Overall, this study offers an important perspective on lipid parameters and ADRD risk.

## CONFLICT OF INTEREST STATEMENT

The author declares no conflicts of interest. Author disclosures are available in the supporting information.

## Supporting information



Supporting Information
